# Structure and Mechanism of the Divalent Anion/Na^+^ Symporter

**DOI:** 10.3390/ijms20020440

**Published:** 2019-01-21

**Authors:** Min Lu

**Affiliations:** Department of Biochemistry and Molecular Biology, Rosalind Franklin University of Medicine and Science, 3333 Green Bay Road, North Chicago, IL 60064, USA; min.lu@rosalindfranklin.edu; Tel.: +1-847-578-8357

**Keywords:** membrane protein, anion transporter, sodium symporter, dicarboxylate transporter, substrate recognition, sodium coordination

## Abstract

Integral membrane proteins of the divalent anion/Na^+^ symporter (DASS) family are conserved from bacteria to humans. DASS proteins typically mediate the coupled uptake of Na^+^ ions and dicarboxylate, tricarboxylate, or sulfate. Since the substrates for DASS include key intermediates and regulators of energy metabolism, alterations of DASS function profoundly affect fat storage, energy expenditure and life span. Furthermore, loss-of-function mutations in a human DASS have been associated with neonatal epileptic encephalopathy. More recently, human DASS has also been implicated in the development of liver cancers. Therefore, human DASS proteins are potentially promising pharmacological targets for battling obesity, diabetes, kidney stone, fatty liver, as well as other metabolic and neurological disorders. Despite its clinical relevance, the mechanism by which DASS proteins recognize and transport anionic substrates remains unclear. Recently, the crystal structures of a bacterial DASS and its humanized variant have been published. This article reviews the mechanistic implications of these structures and suggests future work to better understand how the function of DASS can be modulated for potential therapeutic benefit.

## 1. Introduction

**Figure 1:**
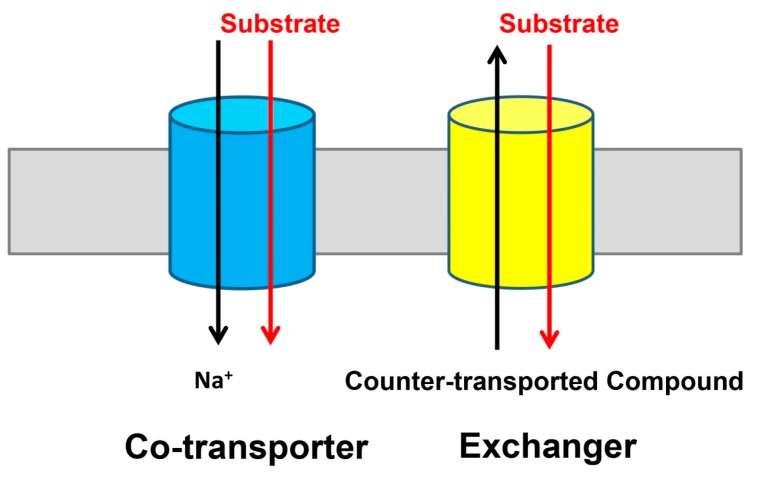
Two modes of transport identified in the divalent anion/Na^+^ symporter (DASS) proteins. Many DASS proteins, including VcINDY and five human DASS transporters, function as Na^+^-dependent co-transporters or symporters (**left**). Whereas, the fruit fly INDY, which is the founding member of the DASS family, appears to be a Na^+^-independent exchanger or antiporter (**right**). For simplicity, the DASS proteins are drawn as cylinders (blue and yellow) and the membrane as a grey rectangle.

Integral membrane proteins from the divalent anion/Na^+^ symporter (DASS) family are found in all domains of life [1,2,3]. They typically move Krebs cycle intermediates or sulfate across cell membranes by dissipating the electrochemical Na^+^ gradient. Specifically, mammalian DASS proteins NaDC1, NaDC3, and NaCT co-transport three or more Na^+^ ions and C_4_-dicarboxylate (such as succinate) or C_6_-tricarboxylate (such as citrate), whereas NaS1 and NaS2 co-transport two or three Na^+^ ions and sulfate [4,5,6,7,8]. Mammalian DASS proteins carry out their function at the plasma membrane of epithelial cells or cells of the central nervous system. The location of and functional difference among the human DASS proteins has been previously reviewed [9] and therefore not discussed here. Additionally, previous phylogenetic analysis has also suggested that the five human DASS transporters can be divided into three sub-groups [9]. The bacterial DASS proteins, by contrast, are located in the cytoplasmic membrane and catalyze the coupled uptake of two or more Na^+^ ions and C_4_-dicarboxylate [10,11,12,13,14]. Although most of the DASS proteins are co-transporters or symporters (Figure 1), some members from the non-vertebrates, including INDY from the fruit fly, function as exchangers and are Na^+^-independent [15,16].

Since the DASS substrates include key intermediates and regulators of energy metabolism, the modulation of DASS activity can profoundly impact fatty acid synthesis, energy expenditure, and life span. For example, a number of mutations in a DASS-encoding gene had been found to nearly double the average adult life-span in fruit flies, likely by promoting a metabolic state that mimics caloric and dietary restriction [17,18]. In addition, the knockdown of the genes encoding NaDC2 and NaCT in worms could decrease their body size and fat content, and/or increase their life span [19,20]. Moreover, the deletion of the gene encoding NaCT protected mice from the adiposity and insulin resistance induced by high-fat feeding and aging [21]. In addition, several loss-of-function mutations in human NaCT have been associated with severe epilepsy and encephalopathy early in life, as well as developmental delay and tooth dysplasia in children [22]. Furthermore, a recent study reported that the loss of NaCT could halt the growth of liver cancer cells, probably by changing both the energy production and cell signaling in these cells [23]. Apart from the di/tricarboxylate substrates, sulfate is one of the most abundant anions in mammalian plasma. As such, mammalian NaS1 and NaS2 have been implicated in regulating sulfate conjugation and the detoxification of xenobiotics [2]. 

Altogether, these studies support human DASS proteins as potentially novel therapeutic targets for tackling diet-induced obesity, type 2 diabetes, kidney stone, and fatty liver, in addition to other metabolic and neurological disorders [1,2,3]. Despite such importance, the molecular mechanism of DASS remained unclear, largely owing to the paucity of structural information on any substrate-bound DASS. Recently, the X-ray structures of a bacterial DASS have been reported, elucidating the transporter architecture as well as the Na^+^- and substrate-binding sites [24,25]. This review discusses these structures in the context of relevant biochemical data and suggests future directions towards illuminating the general principles underlying DASS-mediated transport.

## 2. Structure Determination of DASS

**Figure 2:**
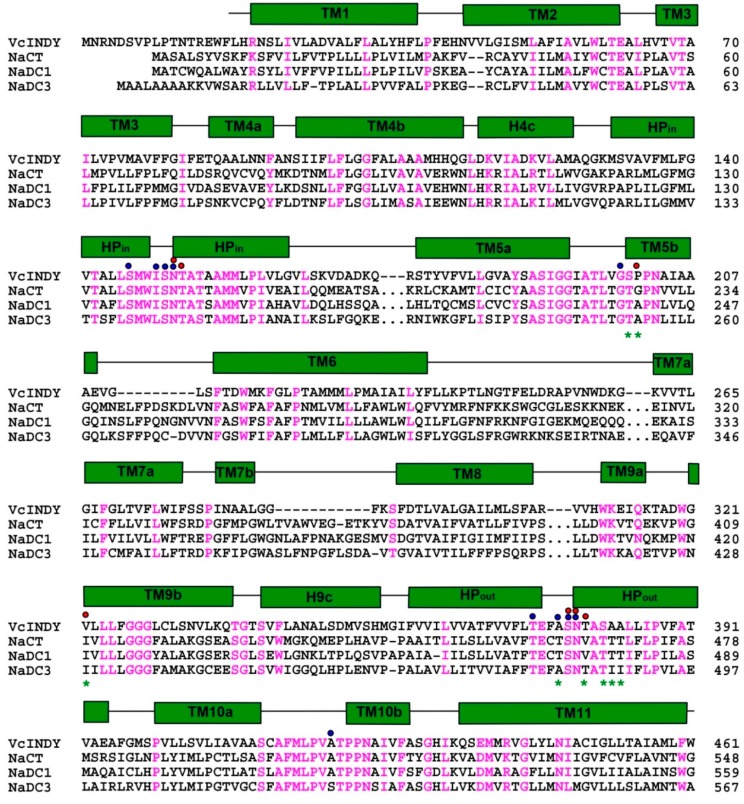
Amino-acid sequence alignment of representative DASS proteins. Residues conserved among VcINDY and three human orthologues are colored magenta, regions of secondary structural elements in VcINDY are outlined, with transmembrane helices shown as green rectangles. Red and blue dots highlight amino acids that bind succinate/citrate and Na_+_, respectively. Positions for the relevant humanizing mutations, which include S200T, P201G, V322I, T379V, A376T, S381T, A382T, and A383T, are marked by green asterisks. For clarity, some residues in the human DASS proteins were omitted and indicated by “…”. Notably, the amino-acid sequence identity between VcINDY and NaCT is 23%, but the degree of sequence conservation in and around the citrate- and Na_+_-binding sites is substantially higher, suggesting that the VcINDY structure provides a useful model for studying the mechanism of NaCT or other human DASS. Sequence alignment was performed by using the program ClustalW.

The molecular structure of any DASS remained unknown until 2012, when the 3.2 Å resolution crystal structure of citrate-bound VcINDY (Figure 2), a DASS from *Vibrio cholerae*, was reported [24]. This structure (PDB 4F35) revealed the transporter architecture and implicated the amino acids in Na^+^- and citrate-binding. However, like other well-characterized bacterial DASS proteins, VcINDY is known to transport succinate and other C_4_-dicarboxylates, rather than citrate, a C_6_-tricarboxylate [14,24]. Furthermore, although VcINDY was suggested to catalyze the co-transport of three Na^+^ ions and C_4_-dicarboxylate [14], only one Na^+^-binding site was observed in the 3.2 Å resolution X-ray structure [24]. Although a second Na^+^-binding site was predicted, no direct structural evidence was found and the assignment of this site was uncertain. Thus, the 3.2 Å structure sheds little light on how VcINDY recognizes substrate and multiple Na^+^ ions.

To address such critical questions, the structure of succinate-bound VcINDY, determined at a resolution of 2.8 Å, was published in 2017 [25]. This structure (PDB 5UL7) elucidates a previously undiscovered Na^+^-binding site in VcINDY as well as how this protein selects for trans-C_4_-dicarboxylate. In the same study, the structure of a citrate-bound VcINDY (PDB 5UL9) as well as those of the succinate- (PDB 5ULD) and citrate-bound MT5 (PDB 5ULE), a humanized variant of VcINDY, were established at 2.8 Å resolution [25]. These crystal structures cast new light on how citrate competitively inhibits VcINDY-mediated succinate transport as well as how a DASS distinguishes between C_4_-dicarboxylate and C_6_-tricarboxylate. In combination with mutagenesis and functional studies, these structures offer a solid framework for understanding how DASS proteins select and transport anionic substrates.

## 3. Overall Structure of VcINDY

**Figure 3:**
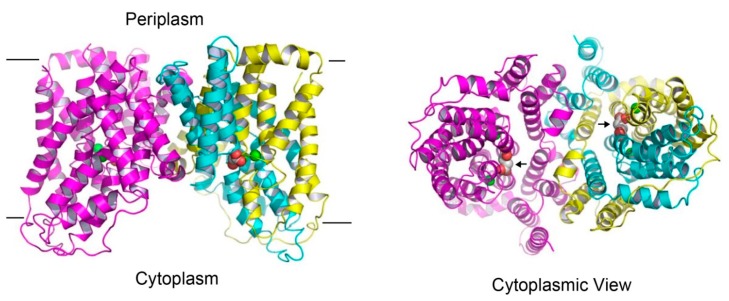
Structure of the succinate-bound VcINDY. Structure of dimeric VcINDY, as viewed from the membrane bilayer (**left panel**). VcINDY is shown in ribbon rendition, the N (amino acids 18-231) and C (amino acids 232-462) domains in one protomer are colored cyan and yellow, respectively, whereas the other protomer is colored magenta. Na^+^ ions (green) and succinate are drawn as spheres. The cytoplasmic view of the VcINDY structure (**right panel**), highlighting the solvent-accessible succinate (black arrows) and buried Na^+^ ions. As such, the crystal structure captures the transporter in the inward-open state. Unless noted otherwise, structural analysis in this review was performed by using the program O and figure was prepared by using the software PyMOL.

The structure of succinate-bound VcINDY [25] reveals a homodimeric arrangement (Figure 3), with each protomer consisting of eleven membrane-spanning helices (named TM1-TM11), two re-entrant helix-turn-helix hairpins (HP_in_ and HP_out_), and two interfacial helices (H4c and H9c). As viewed from the membrane, the VcINDY dimer looks like an inverted bowl with its concave side facing the cytoplasm, thereby allowing for the aqueous solution to reach the midpoint of the membrane. This protein architecture facilitates substrate diffusion to the binding site and it partially solves the problem of translocating anionic substrate across the hydrophobic lipid bilayer, an energetically unfavorable process. The N- and C-domains of VcINDY are similarly folded, despite opposite membrane topology and modest amino-acid sequence similarity [25].

Moreover, VcINDY bears structural resemblance to the dimeric AbgT transporters, MtrF and YdaH [26,27] despite a lack of significant amino-acid sequence similarity. Specifically, the structure of succinate-bound VcINDY can be superimposed onto those of MtrF (PDB 4R1I) and YdaH (PDB 4R0C) to yield rms deviations of 3.1 and 3.5 Å for 294 and 305 Cα atoms, respectively. Moreover, VcINDY bears 18 and 13% amino-acid sequence identity to MtrF and YdaH, respectively. Although most of the DASS proteins, including VcINDY, are co-transporters, the AbgT transporters function as antibiotic efflux pumps and they are exchangers [26,27]. Apparently, the AbgT and DASS proteins constitute a new group of secondary membrane transporters with shared dimeric organization and structural fold, even though they seem to have distinct physiological functions and transport mechanisms. It remains unclear, however, whether the AbgT and DASS proteins arose from convergent or divergent evolution.

## 4. Na^+^-binding Sites in VcINDY

**Figure 4:**
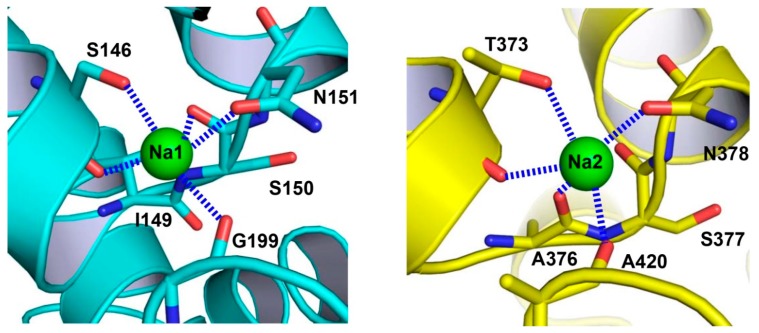
Close-up views of the Na^+^-binding sites in VcINDY. Structure of the Na^+^-binding site in the N domain (**left panel**). The previously unobserved Na^+^-binding site within the C domain (**right panel**). Na^+^ ions are drawn as green spheres and relevant amino acids as stick models. Dashed lines (blue) indicate the coordination interactions.

The binding sites of two Na^+^ ions, designated as Na1 and Na2 (Figure 4), were identified in VcINDY [25]. Despite compelling structural evidence, it is generally a challenge to unambiguously establish the Na^+^-binding sites in protein structures. Therefore, to validate the observed Na^+^-binding sites, one putative cation-coordinating amino acid in Na1 or Na2 was replaced by Ala. Both of these two single mutants exhibited impaired transporter activity and substantially altered Na^+^-dependence of succinate transport, thereby confirming the assigned Na^+^-binding sites [25]. Moreover, most of the Na^+^-binding amino acids are conserved (Figure 2), suggesting that both cation binding sites are shared by the DASS members [25]. Notably, the binding site Na2 can also be found in YdaH [25,27], further supporting the notion that the AbgT and DASS transporters represent a group of membrane transporters with similar structure.

In VcINDY, each Na^+^ ion is penta-coordinated to two amino-acid side-chain and three backbone carbonyl oxygen atoms (Figure 4). The two Na^+^ ions are bound to the pseudo-symmetry-related HP_in_ and HP_out_, and thus Na1 and Na2 are structurally similar [25]. The structures of Na1 and Na2 also resemble those of the Na^+^-binding sites found in other transporters, including AbgT, VcCNT (a Na^+^-dependent concentrative nucleoside transporter), and Glt_Ph_ (a Na^+^-coupled aspartate symporter). All of these binding sites comprise a helix-turn-helix hairpin and a discontinuous helix [27,28,29], thereby defining a class of widespread Na^+^-binding motifs in membrane proteins. By contrast, yet another common Na^+^-binding motif can be found in the transporters with the LeuT-like structural fold [30], which consists of a substantially bent and discontinuous helix in addition to a long but usually continuous helix. 

## 5. Di- and Tri-carboxylate Binding Sites in VcINDY

**Figure 5:**
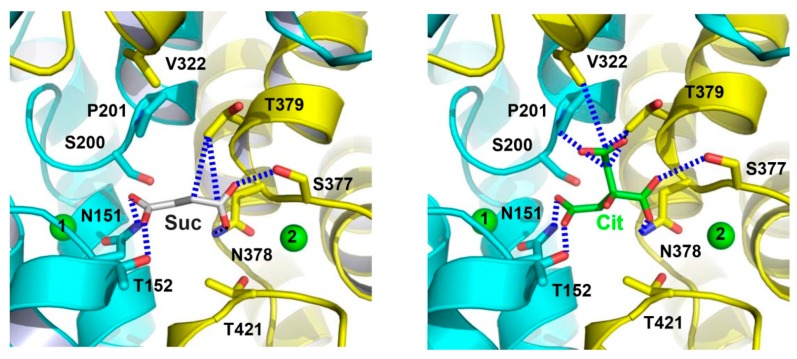
Close-up views of the succinate- and citrate-binding sites in VcINDY. Structure of the succinate-binding site (**left panel**). Detailed view of the citrate-binding site (**right panel**). Succinate (grey), citrate (green) and relevant amino acids are drawn as stick models, whereas the Na^+^ ions are shown as green spheres. Dashed lines (blue) highlight the interactions between VcINDY and succinate or citrate.

Within the Na^+^-binding cleft in VcINDY, the binding sites for succinate and citrate were also observed [25]. Specifically, the bound succinate interacts with VcINDY through H-bonding interactions (Figure 5) and it is partly exposed to the cytoplasm, indicating that the transporter adopts an inward-open conformation (Figure 3). Moreover, the alanine substitutions of several succinate-binding amino acids reduced the binding of succinate to VcINDY and impaired transport function, thereby confirming the biological relevance of the substrate-binding site [24,25]. Notably, the bound succinate adopts an extended conformation, arguing that VcINDY is specific for C_4_-dicarboxylate in a stretched conformation. Since most of the succinate-interacting amino acids are evolutionarily conserved (Figure 2), this preference for trans-dicarboxylate is likely to be shared by the DASS proteins.

The citrate-bound VcINDY is structurally similar to the succinate-bound protein, with the citrate- and succinate-binding sites overlapping substantially, which is consistent with the contention that citrate inhibits the transport of succinate by preoccupying the substrate-binding site in VcINDY, i.e., as a competitive inhibitor. Furthermore, the two co-crystal structures suggest that HP_in_, HP_out_, and the unwound region in TM10 constitute a “trans-dicarboxylate-recognition” module in DASS [25]. This module lacks any protonatable or positively charged amino acids, starkly contrasting the succinate-binding water-soluble proteins, in which Arg and Lys form charge-charge interactions with the bound dicarboxylate [31,32,33,34,35].

In vivo, at least two carboxylates in citrate are deprotonated and negatively charged [14]. In the citrate-bound VcINDY, one carboxylate group makes no contact with the transporter. By contrast, both carboxylates in succinate are stabilized by the H-bonding interactions made with VcINDY. Therefore, the negative charges in citrate appears not fully “neutralized” by its interactions with VcINDY, which may explain why citrate is less effective in inhibiting VcINDY–mediated transport than C_4_-dicarboxylates and why citrate preferably binds to the inward-facing VcINDY [14,25]. 

Previous studies also implied that the coordination of Na^+^ promotes substrate binding to DASS [10,11,12,13]. In VcINDY, the Na^+^ ions in Na1 and Na2 coordinate several succinate-binding amino acids and thus stabilize the conformation of these amino-acid side chains. This arrangement helps to explain why the transport of succinate and Na^+^ is strictly coupled, as they bind to a common subset of amino acids, and the binding or unbinding of one likely affects that of the other [25]. Moreover, the Na^+^ ions may attract negatively charged succinate through long-range electrostatic interactions within the low-dielectric intramembrane environment. Furthermore, the amino ends of four short helices from HP_in_, HP_out_, TM5, and TM10, which possess localized positive dipoles, all point toward the bound succinate. The stabilization of negative charges by the opposing, positive helix dipoles within inverted structural repeats seems to be a common strategy in achieving anion selectivity by membrane proteins [36,37,38,39]. 

## 6. Structures of A Humanized Variant of VcINDY

**Figure 6:**
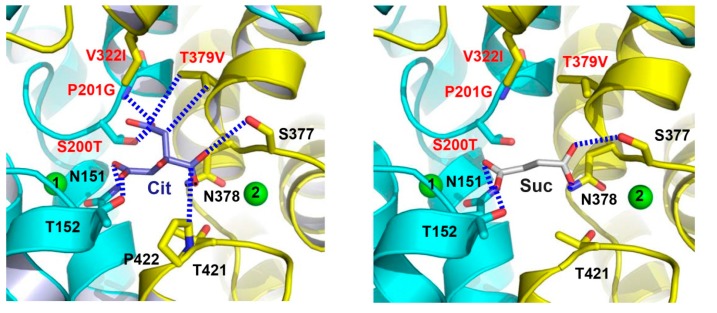
The citrate- and succinate-binding sites in humanized variant of VcINDY. Structure of the citrate-binding site (**left panel**). Close-up view of the succinate-binding site (**right panel**). Citrate (light blue), succinate (grey) and relevant amino acids are drawn as stick models, while the Na^+^ ions are shown as green spheres. Humanizing amino-acid substitutions are highlighted in red. Dashed lines (blue) indicate the interactions between MT5 and citrate or succinate.

To gain new insights into the transport mechanism of human DASS, the structures of a humanized variant of VcINDY in complexes with citrate and succinate were determined to 2.8-Å resolutions [25]. In order to generate this mutant, MT5, eight amino acids surrounding the citrate-binding cleft were replaced by their counterparts in human NaCT (Figure 2), which primarily transports citrate [6]. Although the structure of citrate-bound MT5 remains similar to that of VcINDY, one important difference centers on one carboxylate of the bound citrate (Figure 6). In contrast to that in VcINDY, this carboxylate group from citrate latches onto the amino ends of TM5b and the second helix in HP_out_ in MT5, with its putative negative charge being stabilized by the positive helix dipoles. Since NaCT transports trianionic citrate [6] and MT5 was co-crystallized with citrate at pH~7 [25], the citrate-bound MT5 structure may predict the interactions between NaCT and its bound substrate in vivo.

Interestingly, the succinate-bound structure of MT5 revealed that the humanized variant binds succinate virtually in the same way as VcINDY (Figure 6). Moreover, the transport kinetics for MT5-mediated succinate uptake is also similar to those of VcINDY [25]. However, citrate inhibited the MT5-meditated uptake of succinate much more effectively than it did on VcINDY [25]. In accordance with this observation, MT5 seemed to interact with citrate more strongly within the crystals than VcINDY. Although no appreciable citrate-transporting activity in MT5 was detected, the structure of citrate-bound MT5 likely recapitulates the substrate-binding properties of NaCT to a significant extent, in light of the amino-acid sequence similarity between VcINDY and NaCT, as well as the homolog swap mutations carried by MT5 (Figure 2). It remains unclear, on the other hand, why MT5 failed to transport citrate in vitro despite its higher affinity for citrate than that of VcINDY [25]. One plausible explanation would be that MT5 still lacks key structural elements that somehow enable NaCT to transport citrate more effectively than MT5, which may be found through inspection of the amino-acid sequence alignment (Figure 2) and further mutagenesis study. 

## 7. Substrate Recognition By DASS and Other anion Transporters

**Figure 7:**
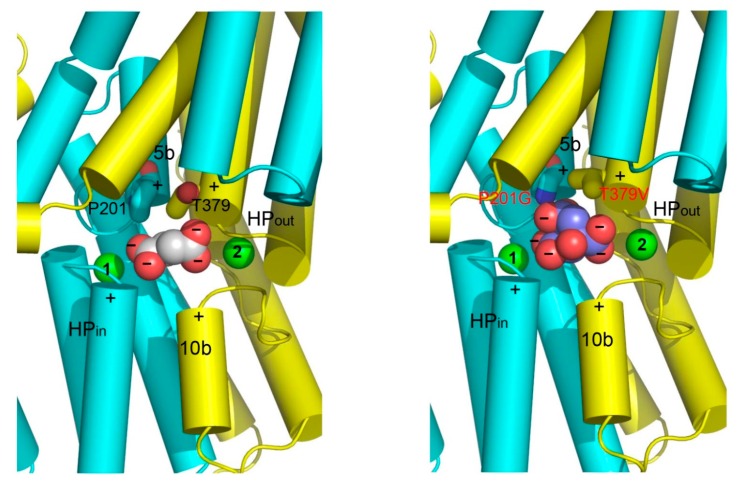
Structural basis for substrate recognition by DASS. The N and C domains in VcINDY (**left panel**) and its humanized variant (**right panel**) are colored cyan and yellow, respectively. Relevant amino acids are drawn as stick models, whereas the bound succinate (**left panel**) or citrate (**right panel**), as well as the Na^+^ ions (green) are shown as spheres. Positive helix dipoles for short helices TM5b and TM10b are highlighted by plus signs, whereas the negatively charged carboxylates in succinate or citrate are indicated by minus signs. The charged state of succinate or citrate is deduced based on the crystallization pH (~7). Both the helix dipoles and Na^+^ appear to contribute to the anion binding. Furthermore, P201 and T379 may enable VcINDY to select for succinate but against citrate. In MT5, P201, and T379 are replaced by Gly and Val, respectively, which may allow the membrane-embedded transporter to bind citrate more strongly than VcINDY.

The structures of VcINDY and its humanized variant suggest that the amino ends of TM5b and the second helix in HP_out_ form a second substrate-recognition module in DASS for differentiating C_6_-tricarboxylate from C_4_-dicarboxylate [25]. In a C_4_-dicarboxylate-specific VcINDY, this module includes a Pro and a Thr (Figure 7), which selects against citrate by pushing away one of its carboxylate groups and likely gives rise to negative charge surplus within the hydrophobic membrane environment. In a C_6_-tricarboxylate-transporting NaCT, however, the Pro and Thr are superseded by Gly and Val, respectively, which enables direct interaction with the same carboxylate group in citrate (Figure 7). Thus, DASS appears to be equipped with two substrate-recognition modules: one selective for trans-C_4_-dicarboxylate and the other for C_6_-tricarboxylate. Na^+^ also contributes to the binding of C_4_-dicarboxylate to DASS by stabilizing the first structural module. Taken together, these structures shed new light on how a DASS recognizes its substrate and offer a new angle to understand protein-mediated anion transport in general [25].

A striking feature of the substrate-binding site in VcINDY is the absence of any positively charged amino acid, i.e., Lys or Arg. Apparently, DASS has evolved such a scheme probably because positively charged amino acids would discourage the binding of Na^+^ in their vicinity due to electrostatic repulsion and/or cause the transporter to bind anionic substrate much too tightly, thereby discouraging the dissociation of substrate from the transporter [25]. Besides DASS, at least three families of transporters with available structures selectively transport anionic substrates: SeCitS and KpCitS from the citrate-sodium symporter family [40,41], Glt_Ph_ and Glt_Tk_ from the excitatory amino acid transporter family [29,42], and NarK and NarU from the nitrate/nitrite porter family [43,44,45]. 

In contrast to VcINDY, all the other three anion transporters utilize positively charged amino acids to bind the negatively charged groups in the substrate. This difference may reflect the distinct substrate-binding sites and/or the coupling mechanisms [25]. For example, an Arg residue in the substrate-binding site of Glt_Ph_ appears critical in determining acidic versus neutral substrate selectivity, since the neutralization of this Arg residue increased the tendency of the transporter to select for neutral rather than acidic substrate [46]. In Glt_Ph_, however, an Asp residue is located in close proximity to this Arg, the former of which may neutralize the positive charge on the Arg side-chain and weaken the electrostatic attraction between the Arg and anionic substrate, thereby facilitating the release of the negatively charged substrate during transport. 

## 8. Elevator-like Mechanism and Future Perspectives

Despite recent progress in the structural studies of VcINDY, great uncertainties remain in the mechanistic understanding of the DASS-mediated anion transport. Particularly, in all the known structures of VcINDY, the substrate-binding site opens into the cytoplasm, i.e., adopting the inward-facing conformation. Therefore, the molecular basis for the interconversion between the inward- and outward-facing conformations, which lies at the heart of the transport mechanism [47,48], remains unclear. Moreover, the structural comparison of the succinate- and citrate-bound VcINDY and its humanized variant gave little insight into how a DASS selects between di/tricarboxylates and sulfate. Furthermore, although modulation of the function of DASS seems to be a viable therapeutic option for battling metabolic and neurological disorders, the mechanism of such modulation is poorly understood.

**Figure 8:**
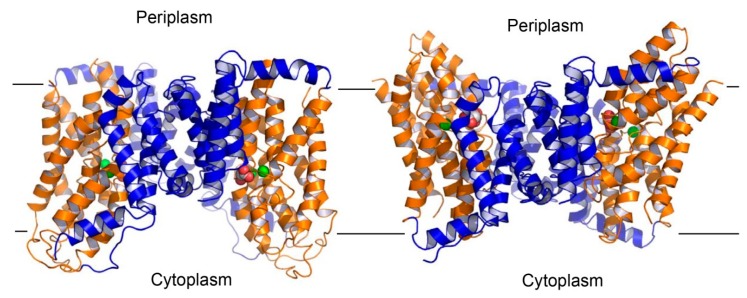
Structural model of the outward-facing VcINDY. Structure of the inward-facing VcINDY as viewed from the membrane bilayer (**left panel**). VcINDY is drawn as a ribbon diagram, with its scaffold (residues 18-128, 250-357) and transport (residues 129-249, 358-462) domains colored blue and orange, respectively. Na^+^ ions (green) and succinate are shown as spheres. The structural model of the outward-facing VcINDY (**right panel**). The orientation and coloring scheme are both the same as in panel A. As the transport domain traverses the membrane, VcINDY alternates between the inward- and outward-facing conformations.

To address these critical questions, future work should be aimed at deciphering the structure of an outward-facing DASS. Previous studies have implied that VcINDY undergoes an elevator-like movement during transport [49]. Such a structural mechanism appears to be widespread in a variety of transporters with diverse physiological function, including the anion transporters SeCitS and Glt_Ph_ [50]. In these transporters, the protein can be divided into the scaffold and transport domains, the latter of which contains the substrate- and cation-binding sites. During transport, the scaffold domain, or the “hoistway”, remains stationary, whereas the transport domain, or the “cabin”, moves up and down, thereby exposing the substrate- and cation-binding sites alternately to either side of the membrane. Based on this concept, a structural model of the outward-facing VcINDY (Figure 8) can be built by using the succinate-bound structure [25]. Despite its usefulness as a starting point for deciphering the transport mechanism, this outward-facing model of VcINDY needs to be further modified through additional biochemical and/or structural studies, since the transporter likely interacts with its substrate somewhat differently in distinct conformations [41].

This elevator-like mechanism also predicts that any compounds that can glue the transport and scaffold domains of DASS together can serve as effective inhibitors of the transporter. Thus, the structural model of outward-facing VcINDY alongside the inward-facing structure may be useful for designing such inhibitors. These compounds likely act as allosteric inhibitors of DASS and they are presumably different from what have been studied previously, as prior work appears to focus on those chemicals that target the Na^+^- and substrate-binding sites in the human DASS transporters [51,52]. Needless to say, these allosteric inhibitors can vastly expand the scope for the development of potentially useful therapeutics that target the human DASS proteins. Furthermore, to gain further insights into the mechanism of the sulfate-transporting DASS proteins, including human NaS1 and NaS2, future work should also include the structure determination of a sulfate-transporting DASS. Although challenging, these studies will advance our understanding of how a DASS transports key metabolites inside cells and how its function can be modulated. Such knowledge promises to inform the design of new pharmaceuticals to prevent and treat the DASS-associated diseases. 

## Abbreviations

DASS	Divalent Anion/Sodium Symporter
NaDC1	Na^+^-dependent DiCarboxylate symporter1
NaDC3	Na^+^-dependent DiCarboxylate symporter3
NaCT	Na^+^-dependent Citrate Transporter
INDY	I’m Not Dead Yet
NaS1	Na^+^-dependent Sulphate symporter1
NaS2	Na^+^-dependent Sulphate symporter2
VcINDY	*Vibrio Cholerae* I’m Not Dead Yet (a divalent anion/sodium symporter from *Vibrio cholerae*)
